# Associations between body composition and bone loss in early postmenopausal women

**DOI:** 10.1093/jbmr/zjaf125

**Published:** 2025-09-11

**Authors:** Marina Vilar Geraldi, Giulia Gregori, Lisa Johansson, Ulrika Hjertonsson, Emma Brättemark, Mattias Lorentzon

**Affiliations:** Sahlgrenska Osteoporosis Centre, Department of Internal Medicine and Clinical Nutrition, Institute of Medicine, University of Gothenburg, Gothenburg, Gothenburg SE-413 45, Sweden; Sahlgrenska Osteoporosis Centre, Department of Internal Medicine and Clinical Nutrition, Institute of Medicine, University of Gothenburg, Gothenburg, Gothenburg SE-413 45, Sweden; Sahlgrenska Osteoporosis Centre, Department of Internal Medicine and Clinical Nutrition, Institute of Medicine, University of Gothenburg, Gothenburg, Gothenburg SE-413 45, Sweden; Department of Orthopaedics, Sahlgrenska University Hospital, 431 80 Mölndal, Sweden; Sahlgrenska Osteoporosis Centre, Department of Internal Medicine and Clinical Nutrition, Institute of Medicine, University of Gothenburg, Gothenburg, Gothenburg SE-413 45, Sweden; Sahlgrenska Osteoporosis Centre, Department of Internal Medicine and Clinical Nutrition, Institute of Medicine, University of Gothenburg, Gothenburg, Gothenburg SE-413 45, Sweden; Sahlgrenska Osteoporosis Centre, Department of Internal Medicine and Clinical Nutrition, Institute of Medicine, University of Gothenburg, Gothenburg, Gothenburg SE-413 45, Sweden; Geriatric Medicine, Institute of Medicine, Sahlgrenska Academy, Sahlgrenska University Hospital, 431 80 Mölndal, Sweden

**Keywords:** postmenopausal women, BMD, body composition, lean mass, fat mass, bone loss, Osteoporosis

## Abstract

The early postmenopausal period is characterized by rapid bone loss, accompanied by a decline in lean mass and an increase in fat mass, highlighting the importance of understanding how these changes influence bone health. This study aimed to assess the cross-sectional and longitudinal associations between body composition and bone characteristics in early postmenopausal women using linear mixed models for repeated measures. A total of 223 Swedish women, aged 50-60 and within 1-4 yr postmenopause, were followed for 2 yr as part of the ELBOW II clinical trial. Body composition—body weight, appendicular lean mass (ALM), and fat mass—was assessed by DXA. Bone outcomes included areal BMD at the TH, FN, LS (DXA), as well as tibia bone microarchitecture and volumetric BMD (vBMD), measured by HR-pQCT. Higher baseline body weight, BMI, fat mass, and ALM were significantly associated with greater cortical area, cortical vBMD, and total vBMD. Baseline body weight, BMI, and fat mass, but not ALM, were positively associated with TH BMD. Longitudinally, increases in ALM were significantly associated with favorable changes in TH BMD, LS BMD, total vBMD, trabecular bone volume fraction, and cortical area. Changes in body weight and BMI were associated with multiple bone outcomes, while fat mass change was linked only with cortical area. In exploratory group comparisons, women with low baseline fat mass (28.14%) and greater ALM loss (∆% ALM: −2.87 kg) experienced 2.4-fold and 5.2-fold greater reductions in TH BMD and tibia total vBMD, respectively, compared to those with high fat mass and maintained ALM. These findings underscore the importance of maintaining or increasing lean mass and preserving overall body weight to mitigate bone loss and reduce skeletal fragility in early postmenopausal women.

## Introduction

Osteoporosis is a skeletal condition common in the elderly, characterized by low BMD and impaired bone microstructure, leading to increased risk of fractures.^1^ Notably, each SD decrease in BMD is associated with approximately a 2-fold increase in fracture risk.^2^ Bone mineral density declines with age, and women are particularly vulnerable due to the abrupt hormonal changes that occur during the menopause transition.^3^ The first few years following menopause represent a critical period of accelerated bone loss, driven by increased bone turnover and a negative remodeling balance.^4^

Body composition undergoes significant changes during menopause, including accelerated gains in fat mass and losses in lean mass. These changes are well-documented consequences of the menopausal transition and may have important implications for bone health.^5^ While body weight is highly correlated with BMD, the relative contribution of body fat and lean mass—along with their changes over time—remain complex and a subject of ongoing debate.^6^ While several studies have explored the relationship between body composition and BMD during the menopause transitions, most have been cross-sectional in design and yielded inconsistent findings. Some have reported positive associations between lean mass and BMD, whereas others have found no significant relationship.^7–10^ Similarly, the associations between fat mass and BMD have also varied across studies.^8^^,^^11^^,^^12^

Most previous studies have been cross-sectional in design and have relied primary on areal BMD measurements obtained by DXA, with limited evaluation of bone microarchitecture. The aims of this study were to examine the association between body composition and bone characteristics—including microstructure, density, and geometry—in early postmenopausal women, by (1) evaluating whether baseline values of body composition predict subsequent changes in bone parameters, and (2) assessing whether changes in body composition over time are associated with concurrent changes in bone characteristics.

## Materials and methods

### Data source and cohort population

ELBOW II was a 2-yr, double-blind, randomized, placebo-controlled, single-center clinical trial conducted in the greater Gothenburg area in southwestern Sweden. The purpose of the study was to evaluate whether daily supplementation with *Limosilactobacillus reuteri* ATCC PTA 6475 vs placebo could reduce early postmenopausal bone loss. However, the results showed that the probiotic supplementation had no effect on bone loss or bone turnover over the 2-yr treatment period (Table S1).^13^

The cohort consisted of 239 early postmenopausal women who had stopped menstruating within the last 1-4 yr and had serum 25OHD levels greater than 25 nmol/L. Subjects were excluded if they had a T-score of less than −2.5 SD of BMD combined with a 10-yr probability of major osteoporotic fracture of 20% or more, according to the fracture risk assessment tool FRAX;^14^ severe osteoporosis defined as a T-score less than −3.0 in either the TH, FN, or LS; and/or vertebral fracture diagnosed using lateral spine imaging with DXA; previous use of antiresorptive therapy, including systemic hormone therapy (estrogen), bisphosphonates, strontium ranelate, or denosumab; and use of corticosteroid. The intervention involved capsules with *L. reuteri* in two doses, either 5 × 10^8^ (low dose) or 5 × 10^9^ (high dose) colony-forming units, taken twice daily or a placebo. All capsules also included 200 UI of cholecalciferol.^13^

**Figure 1:**
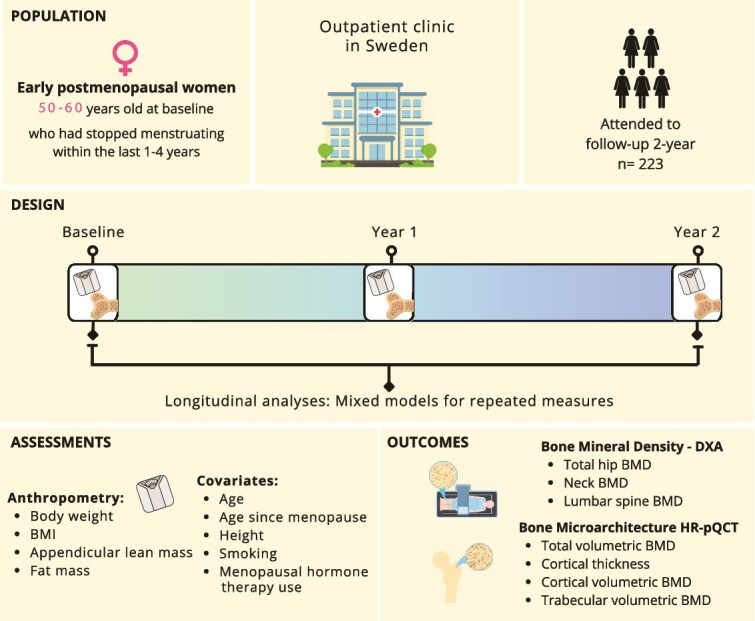
Population, study design summary, assessments and outcomes.

The exclusion and inclusion criteria, study procedures, and outcomes were registered at ClinicalTrials.gov prior to the study’s initiation (registration number: NCT04169789). The study was approved by the Swedish Ethical Review Authority (Dnr: 2019-03740). Written informed consent was obtained from all participants. Women completed questionnaires regarding smoking habits, medical and drug history, and underwent assessments of body composition, bone densitometry, microstructure, geometry, and volumetric BMD (vBMD). Only participants with valid assessments at baseline, 1-yr, and 2-yr visits were included in the analysis (Figure 1).

### Bone microstructure, density, and geometry measurements

Assessments of BMD of the TH and LS were conducted using a GE Lunar iDXA (GE Lunar). The variability in BMD measurements, evaluated in a sample of 8 men and 24 women, yielded coefficients of variation (CVs) of 0.48% for the TH, and 0.73% for the LS. Vertebral fractures (VF) were diagnosed and characterized using the Genant semiquantitative method^15^ using lateral DXA spine imaging as described previously.^16^ Women with grade 2-3 VF were excluded.

Volumetric BMD and bone microstructure were assessed in the distal tibia using HR-pQCT (XtremeCT, Scanco Medical AG) following a previously established protocol.^17^ After image processing, the following variables were derived: total volumetric BMD (vBMD, mg cm^−3^), cortical thickness (Ct.Th, mm), cortical volumetric BMD (vBMD, mg cm^−3^), and trabecular bone volume to total volume fraction (BV/TV, %). The coefficients of variation at our facility, determined in a cohort of 30 women aged 75-80 yr, were 0.2% for total vBMD, 0.5% for cortical thickness, 0.3% for cortical vBMD, and 0.5% for trabecular bone volume fraction.^18^

### Anthropometry analysis

Body weight and height were measured by standard anthropometric techniques. Body mass index was calculated as body weight (kg)/height (m).^2^ Appendicular lean mass (ALM) and fat mass were measured using iDXA.

### Statistical analyses

Categorical variables are presented by number and percentage. Quantitative variables were summarized using the mean ± SD if normally distributed, or otherwise as median with the Interquartile Range (IQR). Comparisons of characteristics over the 2 yr were made by repeated measures ANOVA with Bonferroni post hoc test.

All analyses were conducted using SAS (SAS Institute Inc.). Linear mixed models for repeated measures (MMRM) were applied to examine associations between body composition variables and skeletal outcomes over time. For each body composition measure, two separate models were estimated: (1) using baseline body composition as the main predictor and (2) using body composition at time-varying values. Models included visit as a fixed effect and specified an unstructured covariance matrix to account for within-subject correlations. Although we acknowledge that the exact time interval between visits may vary slightly across individuals, assessments were scheduled at standardized intervals with minimal variation. Interaction *p*-values were used to evaluate potential differences in associations over time. All models were adjusted for baseline age, smoking status, years since menopause, menopausal hormone therapy use, height, visit, treatment group, and the baseline value of the respective skeletal outcome.

The post-hoc statistical power was calculated for the multiple linear regression analysis, based on an *R*^2^ of 0.10, alpha probability level 0.01, the use of 5 predictor variables, and 223 included participants, and resulted in 93% statistical power.

## Results

### Characteristics of study participants

**Figure 2:**
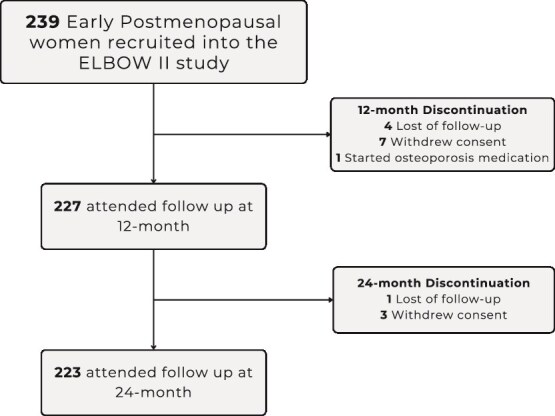
Participant flowchart.

In total, 223 participants completed all study visits and were included in this analysis (Figure 2). At baseline, the median (IQR) age of participants was 55.0 (3) yr, the mean ± SD duration since menopause was 2.2 ± 0.1 yr, and the median BMI was 24.2 (5.8) kg/m^2^. Participants’ characteristics at baseline, 1-yr, and 2-yr follow-up, and relative changes of body composition are shown in Table 1. A decline in all six bone parameters measured was observed during the 2 yr (*p* < .001).

**Table 1:** Baseline characteristics and 1- and 2-yr follow-up of BMD and microstructure, and body composition in early postmenopausal women.

	**Baseline**	**Year 1**	**Year 2**	**∆% 2-yr**	
	*n* = 223	*n* = 223	*n* = 223	*n* = 223	*p*-value
**Characteristics**					
** Age, yr**	55.00 (3)	56.00 (3)	57.00 (3)	–	–
** Years since menopause, yr**	2.23 ± 0.07	3.23 ± 0.07	4.23 ± 0.07	–	–
** Current smoker, *n* (%)**	9 (4.0)	9 (4.0)	9 (4.0)	–	–
**Anthropometry**					
** Height, cm**	166.78 ± 6.82^a^	166.66 ± 6.85^b^	166.64 ± 6.88^b^	−0.05 (0.31)	**<.001**
** Body mass, kg**	66.5 (18.30)	66.80 (19.30)	66.40 (19.20)	0.49 (5.04)	.07
** BMI, kg/m^2^**	24.20 (5.8)^a,b^	24.00 6.0)^a^	24.30 (6.4)^b^	0.90 (5.22)	**.03**
** Percentage body fat, %**	36.26 ± 7.85^a^	36.23 ± 7.98^a^	36.72 ± 7.94^b^	1.73 (7.52)	**<.001**
** Fat mass, kg**	23.49 (14.28)^a^	23.53 (14.15)^a^	24.32 (14.02)^b^	3.10 (12.32)	**.006**
** Appendicular lean mass, kg**	6.58 (0.98)^a^	6.56 (1.00)^b^	6.49 (0.98)^a,b^	0.07 (4.23)	**.02**
**Bone mineral density and microstructure**					
** Total hip BMD, g/cm^2^**	0.96 (0.15)^a^	0.94 (0.16)^b^	0.93 (0.16)^c^	−2.25 (2.80)	**<.001**
** Lumbar spine BMD, g/cm^2^**	1.11 (0.21)^a^	1.11 (0.21)^b^	1.09 (0.20)^c^	5.26 (44.52)	**<.001**
** Tibia total vBMD, mg/cm^3^**	282.5 (62.6)^a^	280.10 (63.0)^b^	274.85 (62.3)^c^	−2.06 (2.60)	**<.001**
** Tibia trabecular BV/TV, %**	0.1285 (0.04)^a^	0.1295 (0.04)^a^	0.1280 (0.04)^b^	−0.65 (2.78)	**<.001**
** Tibia cortical vBMD, mg/cm^3^**	850.10 (73.1)^a^	843.25 (70.5)^b^	836.30 (67.8)^c^	−1.48 (1.91)	**<.001**
** Tibia cortical area, mm**	114.9 ± 20.31^a^	112.67 ± 20.59^b^	110.92 ± 20.44^c^	−3.14 (4.21)	**<.001**

Values are expressed as median (IQR), mean ± SD or percentage (%), as stated. *p*-values were obtained using repeated measures ANOVA and Bonferroni post hoc analysis. Bold values denote statistical significance.

^a-c^Different letters indicate significant differences (*p* < .05) between time points (baseline, year 1, and year 2).

Abbreviations: BV/TV, trabecular bone volume to total volume fraction; vBMD, volumetric BMD.

### Associations with baseline body composition

Associations between baseline body composition, BMD and bone microstructure are presented in Table 2. Higher baseline values of body weight, BMI, ALM, and fat mass were significantly associated with greater cortical area, cortical vBMD, and total vBMD. For areal BMD outcomes, baseline body weight, BMI, and fat mass were positively associated with TH BMD, but not with LS or FN BMD. Baseline ALM was not significantly associated with any DXA-based BMD measures. Associations with trabecular BV/TV were non-significant for all baseline body composition measures.

**Table 2:** Associations between body composition variables and bone outcomes.

**Outcome**	**Body weight**		**BMI**		**Fat mass**		**ALM**	
	**BL β (95% CI)** ***p*-value**	**CHG β (95% CI)** ***p*-value**	**BL β (95% CI)** ***p*-value**	**CHG β (95% CI)** ***p*-value**	**BL β (95% CI)** ***p*-value**	**CHG β (95% CI)** ***p*-value**	**BL β (95% CI)** ***p*-value**	**CHG β (95% CI)** ***p*-value**
**Total hip BMD**	0.035 (0.006; 0.064)**.019**	0.114 (0.036; 0.192)**.0043**	0.031 (0.005; 0.058)**.019**	0.113 (0.044; 0.181)**.0013**	0.037 (0.011; 0.063)**.0059**	0.050 (−0.015; 0.115).13	0.007 (−0.019; 0.032).60	0.130 (0.069; 0.191)**<.0001**
**Neck hip BMD**	0.042 (0.003; 0.081)**.034**	−0.043 (−0.150; 0.064).43	0.038 (0.003; 0.072)**.033**	−0.023 (−0.118; 0.071).63	0.040 (0.005; 0.075)**.027**	−0.067 (−0.156; 0.022).14	0.016 (−0.018; 0.051).36	0.048 (−0.036; 0.133).26
**Lumbar spine**	−0.002 (−0.030; 0.026).90	0.071 (−0.017; 0.160).11	−0.002 (−0.027; 0.023).90	0.071 (−0.007; 0.149).08	0.004 (−0.022; 0.029).77	0.056 (−0.018; 0.130).14	−0.011 (−0.036; 0.014).39	0.091 (0.022; 0.159)**.010**
**Total vBMD**	0.026 (0.009; 0.042)**.0028**	0.049 (0.004; 0.095)**.034**	0.023 (0.008; 0.038)**.0030**	0.048 (0.008; 0.089)**.019**	0.023 (0.008; 0.038)**.0034**	0.024 (−0.014; 0.061).22	0.018 (0.003; 0.033)**.020**	0.058 (0.023; 0.094)**.0015**
**Trabecular**	0.009 (−0.005; 0.024).21	0.027 (−0.013; 0.068).19	0.009 (−0.004; 0.022).19	0.025 (−0.011; 0.061).18	0.012 (−0.001; 0.025).08	0.002 (−0.032; 0.036).89	−0.001 (−0.014; 0.012).88	0.048 (0.016; 0.080)**.0038**
**Cortical vBMD**	0.046 (0.021; 0.071)**.0004**	0.077 (0.003; 0.151)**.042**	0.041 (0.019; 0.064)**.0004**	0.071 (0.005; 0.136)**.035**	0.034 (0.010; 0.057)**.0048**	0.054 (−0.008; 0.115).09	0.047 (0.025; 0.069)**<.0001**	0.044 (−0.015; 0.102).14
**Cortical area**	0.028 (0.005; 0.052)**.018**	0.088 (0.023; 0.153)**.0079**	0.024 (0.003; 0.045)**.025**	0.086 (0.028; 0.143)**.0035**	0.021 (0.000; 0.042)**.047**	0.056 (0.002; 0.110)**.042**	0.024 (0.002; 0.046)**.033**	0.093 (0.043; 0.144)**.0003**

β-estimate are presented per one unit increase in the standardized version of the explanatory variables. Mixed models for repeated measures (MMRM) were used, with an unstructured covariance matrix for correlation of values over time. For the columns, which start with “BL,” the baseline value of the explanatory variable in question was used. For the columns, which start with “CHG,” the change from baseline values (baseline to 2 yr) of the explanatory variable in question was used and assumes consistent associations over time. The models are adjusted for age, smoking, years since menopause, menopausal hormone therapy use, height, visit, treatment group, and the baseline value of each respective outcome. Bold values denote statistical significance.

Abbreviations: BL, baseline; BV/TV, trabecular bone volume to total volume fraction; CHG, change from baseline; vBMD, volumetric BMD. Bone measurements units: TH BMD, g/cm^2^; neck hip BMD, g/cm^2^; LS BMD, g/cm^2^; tibia total vBMD, g/cm^3^; tibia trabecular BV/TV, %; tibia cortical vBMD, g/cm^3^; tibia cortical area, mm^2^.

Total vBMD showed significant interactions with baseline body weight (*p* = .0014), BMI (*p* = .010), and fat mass (*p* = .0005). Cortical vBMD showed significant interactions with baseline body weight (*p* = .0009), BMI (*p* = .0010), fat mass (*p* = .0022), and ALM (*p* = .0003), suggesting time-dependent effects of these baseline measures on cortical bone density. For cortical area, interactions were significant for baseline body weight and BMI (both *p* = .012), and fat mass (*p* = .0048). No significant interactions were found between baseline body composition and visit for DXA-derived outcomes (TH, FN, and LS BMD) or trabecular BV/TV (Table S2).

### Associations with time-varying body composition

The beta coefficients for changes from baseline body composition measures were larger in magnitude and/or more statistically significant than those for baseline body composition values (Table 2). Time-varying body weight and BMI were significantly associated with TH BMD, total vBMD, cortical vBMD, and cortical area. Time-varying ALM was significantly associated with increases in TH BMD (β = .130, 95% CI: 0.069-0.191; *p* < .0001), LS BMD (β = .091, 95% CI: 0.022-0.159; *p* = .010), total vBMD (β = .058, 95% CI: 0.023-0.094; *p* = .0015), trabecular BV/TV (β = .048, 95% CI: 0.016-0.080; *p* = .0038), and cortical area (β = .093, 95% CI: 0.043-0.144; *p* = .0003). Time-varying fat mass was significantly associated only with cortical area (β = .056, 95% CI: 0.002-0.110; *p* = .042).

Significant change × visit interactions were observed (Table S1). For TH BMD, changes in body weight (*p* = .039), BMI (*p* = .050), and especially ALM (*p* < .0001) exhibited significant interaction effects. A significant interaction was also found for ALM change and FN BMD (*p* = .0017), despite the lack of a significant main effect. For cortical area, all four body composition variables showed significant interactions with visit: weight (*p* = .0083), BMI (*p* = .0088), fat mass (*p* = .037), and ALM (*p* = .020), indicating that the association between changes in body composition and cortical area varied across timepoints.

### Illustrative group stratification and longitudinal bone outcomes

For illustrative purposes, participants were ranked into tertiles based on baseline fat mass and relative change in ALM (∆%ALM) and categorized into three groups: two extreme groups representing highest and lowest tertiles of both baseline fat mass and ∆%ALM, and one intermediate group comprising mixed combinations of the two variables (Figure S1; Table 3). Group 1 included individuals in the lowest tertile (T1) for both baseline fat mass and ∆%ALM. Group 2 included those in the middle tertile (T2) for both variables, as well as individuals with mixed profiles—T1 for fat mass and T3 for ∆%ALM, or T3 for fat mass and T1 for ∆%ALM. Group 3 consisted of individuals in the highest tertile (T3) for both baseline fat mass and ∆%ALM.

**Table 3:** Relative change (∆%) in BMD and bone microstructure over 2 yr by change in appendicular lean mass and baseline fat mass in early postmenopausal women.

	**Group 1**	**Group 2**	**Group 3**	
	*n* = 28	*n* = 154	*n* = 28	***p*-value**
**Baseline characteristics**				
** Body mass, kg**	59.5 ± 4.78^a^	66.40 (16.30)^b^	79.80 (11.30)^c^	**<.001**
** BMI, kg/m^2^**	21.68 ± 1.30^a^	24.20 (5.7)^b^	28.2 (5.1)^c^	**<.001**
** Percentage body fat, %**	28.14 ± 3.79^a^	36.46 ± 7.12^b^	45.03 ± 3.38^c^	**<.001**
** Fat mass, kg**	16.71 ± 2.38^a^	24.14 (11.36)^b^	34.77 (10.55)^c^	**<.001**
** Appendicular lean mass, kg**	6.39 ± 0.48^a^	6.49 (1.04)^a^	6.98 (0.54)^b^	**.02**
** Total physical activity, MET**	3184.00 (3544.00)	2700.00 (3266.00)	2652.00 (4063.25)	.66
**Relative changes (∆%) over 2 yr**				
***Body mass and composition***				
** ∆% Body mass, kg**	−1.18 ± 2.24^a^	0.32 (4.97)^a^	3.46 ± 6.03^b^	**<.001**
** ∆% Percentage body fat, %**	3.83 ± 6.63	1.41 (7.52)	1.2 (5.21)	.14
** ∆% Fat mass, kg**	2.69 ± 8.09	1.46 ± 9.98	4.00 ± 12.33	.36
** ∆% Appendicular lean mass, kg**	−2.87 (2.28)^a^	0.22 ± 3.13^b^	3.81 (2.99)^c^	**<.001**
***Bone development***				
** ∆% Total hip BMD, g/cm^2^**	−3.59 ± 2.15^a^	−2.25 (2.72)^a,b^	−1.50 ± 2.94^b^	**.004**
** ∆% Lumbar spine BMD, g/cm^2^**	−2.84 ± 2.26	−1.98 ± 3.00	−2.03 ± 3.37	.32
** ∆% Tibia total vBMD, mg/cm^3^**	−3.33 ± 2.28^a^	−2.15 (2.39)^a^	−0.64 ± 1.87^b^	**<.001**
** ∆% Tibia trabecular BV/TV, %**	−2.05 ± 2.71^a^	−0.68 (2.65)^a^	0.26 ± 2.18^b^	**.002**
** ∆% Tibia cortical vBMD, mg/cm^3^**	−1.91 ± 1.49	−1.59 (1.79)	−1.08 ± 1.47	.12
** ∆% Tibia cortical area, mm^2^**	−4.59 ± 4.19^a^	−3.23 (4.19)^a^	−1.19 ± 3.44^b^	**.004**

Values are median (IQR) or mean ± SD. One-way ANOVA analysis was used to determine statistical significance between groups (*p* < .05) and Bonferroni post-hoc test (*p* < .05). Bold values denote statistical significance.

^a-c^Different letters mean *p* < .05 between groups.

Participants were ranked into tertiles based on baseline fat mass and relative change in ALM (∆%ALM) and categorized into three groups: two extreme groups representing higher and lower tertiles of baseline fat mass and ∆%ALM, and an intermediate group comprising mixed combinations of fat mass and ∆%ALM (Figure S1).

Group 1: Participants in tertile 1 for both baseline fat mass and ∆% ALM.

Group 2: Participants in tertile 2 for both variables or those with mixed combinations, including, tertile 1 for baseline fat mass and tertile 3 for ∆% ALM, or tertile 3 for baseline fat mass and tertile 1 for ∆% ALM.

Group 3: Participants in tertile 3 for both baseline fat mass and ∆% ALM.

Tertiles description: For fat mass, tertile 1 included participants with fat mass ranging from 6.80 to 20.00 kg, tertile 2 ranged from 20.06 to 29.35 kg, and tertile 3 ranged from 29.35 to 58.52 kg. For relative changes in ALM, tertile 1 included participants with changes ranging from −9.77% to −1.33%, tertile 2 ranged from −1.29% to 1.42%, and tertile 3 ranged from 1.44% to 15.73%.

Abbreviations: ∆%, relative change; ALM, Appendicular lean mass; BV/TV, trabecular bone volume to total volume fraction; vBMD, volumetric BMD.

Women in Group 3, who had higher baseline body weight, BMI, fat mass, and ALM, along with greater increases in ∆% body weight and ALM over the following 2 yr, exhibited significantly lower bone loss in terms of more favorable ∆% TH BMD, ∆% tibia total vBMD, ∆% tibia trabecular BV/TV, and ∆% tibia cortical area compared to women in Group 1.

## Discussion

In this longitudinal study of early postmenopausal women, both baseline and time-varying body composition measures were positively associated with skeletal outcomes, although the strength and significance of these associations differed by specific bone site. Higher baseline body weight, BMI, fat mass, and ALM were positively associated with cortical area, cortical vBMD, and total vBMD, while associations with areal BMD were limited to body weight, BMI, and fat mass, and were not observed for LS. Baseline ALM was not associated with any DXA-derived BMD measures, and no baseline body composition variable was significantly associated with trabecular bone volume fraction. Time-varying body composition variables—particularly ALM—showed stronger and more consistent associations with bone outcomes. Increases in ALM were significantly associated with improvements in TH, LS, total vBMD, trabecular BV/TV, and cortical area. Time-varying body weight and BMI were also significantly associated with TH BMD, total vBMD, cortical vBMD, and cortical area, while fat mass was only associated with cortical area. Interaction analyses revealed that several of these associations varied over time. Baseline body composition measures, especially weight, BMI, and fat mass, showed significant interactions with visit for total vBMD, cortical vBMD, and cortical area, suggesting time-dependent predictive effects. Changes in ALM, weight, and BMI showed significant interactions with visit, particularly for hip BMD and cortical geometry, indicating that the influence of body composition on bone may evolve during the postmenopausal transition. These findings underscore the dynamic and compartment-specific nature of the relationship between body composition and bone health and highlight the importance of considering both the timing and type of body composition change in evaluating skeletal outcomes.

Besides prescription-based treatments, evidence-based strategies for managing the menopausal transition emphasize the importance of maintaining a healthy body composition. This includes engaging in regular physical activity, consuming a nutrient-rich diet, and avoiding smoking.^19^ In addition, the combined supplementation of calcium and vitamin D has been found to benefit postmenopausal women, including the prevention of bone loss, reduction of bone turnover, and in the oldest population, prevention of hip fractures associated with osteoporosis.^20^

Body weight and BMI are positively correlated with BMD both at baseline and when modeled as time-varying exposures. However, the contributions of individual components of soft tissue—specifically body fat and muscle mass—add complexity to the relationship between body composition and bone health. The relative impact of these two components on bone characteristics has been widely debated, as it depends on various factors, including gender, ethnicity, age, menopausal status, hormone therapy, corticosteroid use, skeletal site, and other variables. In our longitudinal analysis, increases in ALM were strongly and consistently associated with improvements in bone outcomes, including TH and LS BMD, total vBMD, trabecular BV/TV, and cortical area, whereas changes in fat mass were associated only with cortical area. In contrast, most previous studies have focused on cross-sectional associations to investigate their influence on pre- or postmenopausal women.^21–27^ Some studies have found that both fat and lean mass are significant predictors of BMD,^6^^,^^28^ with lean mass being more important in premenopausal women and fat mass more significant in postmenopausal women.^25^^,^^29^^,^^30^ However, other studies have found that only lean mass is associated with BMD in premenopausal and postmenopausal women.^7^^,^^8^ The importance of different body composition components in preventing bone loss was evaluated by a meta-analysis, which showed that lean mass exerts a greater effect in premenopausal women; however, both lean and fat mass had a similar impact in postmenopausal women.^9^

Longitudinal studies from the Women’s Health Across the Nation (SWAN) examined changes in body composition during the menopause transition.^5^^,^^31^ Greater lean mass loss was associated with lower BMD in the FN, while greater fat mass gain was linked to higher BMD in both FN and LS. However, both greater lean mass loss and greater fat mass gain during the menopause transition were associated with an increased risk of subsequent fractures, suggesting that changes in body composition during menopause may contribute to fracture risk through mechanisms beyond BMD.^31^

Women experience the greatest rate of BMD loss starting one year before the final menstrual period, followed by a deceleration (but not a complete cessation) of loss approximately 2 yr after menopause.^32^ Early postmenopausal bone loss is associated with estrogen deficiency and changes in body composition. Increases in fat mass and losses of lean mass occur during the menopause transition and persist for up to 2 yr after the final menstrual period.^5^ Several mechanisms may underlie the relationship between body composition and bone mass, including mechanical forces acting on the skeleton, physical activity, and various nutritional, genetic, and hormonal factors that contribute to muscle and bone loss.^33^ Skeletal muscle supports bone health not only by generating mechanical loading forces on the skeleton but also through the secretion of paracrine and endocrine factors that influence bone remodeling.^34^ Adipose tissue may have both beneficial and detrimental effects on bone. Increased fat can enhance BMD through greater mechanical loading and peripheral aromatization of androgens to estrogens. On the other hand, adipose tissue is a source of pro-inflammatory cytokines that promote osteoclastogenesis and are negatively associated with BMD.^35^

In an analysis done for illustrative purposes of extreme groups, we found that early postmenopausal women in group 3 (tertile 3 for both baseline fat mass and ∆% ALM), that had a greater increase in ALM (+3.81 (2.99) kg), and higher baseline fat mass (45.03 ± 3.38%), had ~2.4-fold and ~5.2-fold less loss of TH BMD and tibia total vBMD over 2 yr, respectively, compared with those in the group 1 (tertile 1 for both baseline and ∆% ALM). Similarly, a positive relationship between lean mass, fat mass and BMD was found in middle-aged individuals, though these relationships were attenuated or absent in those with higher BMI, and after a 6-yr follow-up, changes in lean mass were found to be a stronger determinant of BMD than fat mass.^36^^,^^37^ However, delineating the independent contribution of ALM and fat mass on bone is not straightforward because these two components are correlated, making it difficult to separate their individual contributions.^6^ The menopause transition is characterized by changes in reproductive hormones, energy intake, and expenditure, which may promote weight gain, increased fat mass, and reduced ALM, thereby contributing to cardiometabolic disease risk.^38^ The TEMPO diet trial showed that postmenopausal women with obesity who underwent severe energy restriction experienced approximately twice the weight and fat loss compared to those who followed moderate energy restriction. These women also lost about 1.5 times more lean mass and 2.5-fold more TH BMD.^39^ While effective for obesity treatment, energy restriction in postmenopausal women, particularly those with osteopenia or osteoporosis, requires careful consideration.

Strengths of our study include the extensive methodology to evaluate bone characteristics, using both DXA and HRpQCT. While most research relies solely on areal BMD from DXA scans to assess bone strength, our study incorporated additional HRpQCT data, allowing a more detailed evaluation of bone density and microarchitectural parameters. Limitations include the homogeneous study population, composed predominantly of Swedish women, which may limit the generalizability of the results to other races and ethnicities. Additionally, the cohort had a short follow-up period and limited changes in variables.

In conclusion, this longitudinal study of early postmenopausal women highlights the important role of body composition in predicting bone development. Higher baseline body weight, fat mass, and BMI, as well as increases in ALM over time, were associated with more favorable bone outcomes, particularly in cortical density, geometry, and total vBMD. These findings suggest that maintaining or increasing ALM and preserving overall body weight may help mitigate skeletal fragility during the early postmenopausal years.

## Supplementary Material

Supplementary_Material_zjaf125

## Data Availability

Data cannot be made publicly available for ethical and legal reasons. Such information is subject to legal restrictions according to national legislation. Specifically, in Sweden confidentiality regarding personal information in studies is regulated in the Public Access to Information and Secrecy Act (SFS 2009:400). The data underlying the results of this study might be made available upon request, after an assessment of confidentiality. There is thus a possibility to apply to get access to certain public documents that an authority holds. In this case, the University of Gothenburg is the specific authority responsible for the integrity of the documents with research data. Questions regarding such issues can be directed to the head of the Institute of Medicine, Sahlgrenska Academy, University of Gothenburg, Gothenburg, Sweden. Contact information can be obtained from medicin@gu.se.

## References

[1] Lorentzon M, Johansson H, Harvey NC, et al. Osteoporosis and fractures in women: the burden of disease. Climacteric. 2022;25(1):4-10. 34319208 10.1080/13697137.2021.1951206

[2] Kanis JA, Harvey NC, Johansson H, et al. A decade of FRAX: how has it changed the management of osteoporosis? Aging Clin Exp Res. 2020;32(2):187-196. 32043227 10.1007/s40520-019-01432-y

[3] Khosla S, Melton LJ, Riggs BL. The unitary model for estrogen deficiency and the pathogenesis of osteoporosis: is a revision needed? J Bone Miner Res. 2011;26(3):441-451. 20928874 10.1002/jbmr.262 PMC3179298

[4] Sowers MR, Zheng H, Greendale GA, et al. Changes in bone resorption across the menopause transition: effects of reproductive hormones, body size, and ethnicity. J Clin Endocrinol Metab. 2013;98(7):2854-2863. 23666961 10.1210/jc.2012-4113 PMC3701268

[5] Greendale GA, Sternfeld B, Huang M, et al. Changes in body composition and weight during the menopause transition. JCI Insight. 2019;4(5):e124865. 10.1172/jci.insight.124865 PMC6483504 30843880

[6] Ho-Pham LT, Nguyen ND, Lai TQ, Nguyen TV. Contributions of lean mass and fat mass to bone mineral density: a study in postmenopausal women. BMC Musculoskelet Disord. 2010;11(1):59. 20346165 10.1186/1471-2474-11-59 PMC2867833

[7] Khosla S, Atkinson EJ, Riggs BL, Melton LJ. Relationship between body composition and bone mass in women. J Bone Miner Res. 1996;11(6):857-863. 8725184 10.1002/jbmr.5650110618

[8] Kim JH, Choi HJ, Kim MJ, Shin CS, Cho NH. Fat mass is negatively associated with bone mineral content in Koreans. Osteoporos Int. 2012;23(7):2009-2016. 22006041 10.1007/s00198-011-1808-6

[9] Ho-Pham LT, Nguyen UDT, Nguyen TV. Association between lean mass, fat mass, and bone mineral density: a meta-analysis. J Clin Endocrinol Metab. 2014;99(1):30-38. 24384013 10.1210/jc.2014-v99i12-30A

[10] Yang S, Center JR, Eisman JA, Nguyen TV. Association between fat mass, lean mass, and bone loss: the Dubbo osteoporosis epidemiology study. Osteoporos Int. 2015;26(4):1381-1386. 25572048 10.1007/s00198-014-3009-6

[11] Salamone LM, Glynn N, Black D, et al. Body composition and bone mineral density in premenopausal and early perimenopausal women. J Bone Miner Res. 1995;10(11):1762-1768. 8592954 10.1002/jbmr.5650101120

[12] Park J-H, Song Y-M, Sung J, et al. The association between fat and lean mass and bone mineral density: the healthy twin study. Bone. 2012;50(4):1006-1011. 22306928 10.1016/j.bone.2012.01.015

[13] Gregori G, Pivodic A, Magnusson P, et al. *Limosilactobacillus reuteri* 6475 and prevention of early postmenopausal bone loss. JAMA Netw Open. 2024;7(6):e2415455. 38865129 10.1001/jamanetworkopen.2024.15455 PMC11170297

[14] McCloskey EV, Harvey NC, Johansson H, et al. Fracture risk assessment by the FRAX model. Climacteric. 2022;25(1):22-28. 34319212 10.1080/13697137.2021.1945027

[15] Blake GM, Rea JA, Fogelman I. Vertebral morphometry studies using dual-energy X-ray absorptiometry. Semin Nucl Med. 1997;27(3):276-290. 9224667 10.1016/s0001-2998(97)80029-3

[16] Johansson L, Sundh D, Magnusson P, et al. Grade 1 vertebral fractures identified by densitometric lateral spine imaging predict incident major osteoporotic fracture independently of clinical risk factors and bone mineral density in older women. J Bone Miner Res. 2020;35(10):1942-1951. 32539162 10.1002/jbmr.4108

[17] MacNeil JA, Boyd SK. Improved reproducibility of high-resolution peripheral quantitative computed tomography for measurement of bone quality. Med Eng Phys. 2008;30(6):792-799. 18164643 10.1016/j.medengphy.2007.11.003

[18] Jaiswal R, Zoulakis M, Axelsson KF, et al. Increased bone material strength index is positively associated with the risk of incident osteoporotic fractures in older Swedish women. J Bone Miner Res. 2020;38(6):860-868. 10.1002/jbmr.4816 37088885

[19] Shifren JL, Gass MLS. The North American Menopause Society recommendations for clinical Care of Midlife Women. Menopause. 2014;21(10):1038-1062. 25225714 10.1097/GME.0000000000000319

[20] Liu C, Kuang X, Li K, Guo X, Deng Q, Li D. Effects of combined calcium and vitamin D supplementation on osteoporosis in postmenopausal women: a systematic review and meta-analysis of randomized controlled trials. Food Funct. 2020;11(12):10817-10827. 33237064 10.1039/d0fo00787k

[21] Sipilä S, Törmäkangas T, Sillanpää E, et al. Muscle and bone mass in middle-aged women: role of menopausal status and physical activity. J Cachexia Sarcopenia Muscle. 2020;11(3):698-709. 32017473 10.1002/jcsm.12547 PMC7296268

[22] Juppi H-K, Sipilä S, Cronin NJ, et al. Role of menopausal transition and physical activity in loss of lean and muscle mass: a follow-up study in middle-aged Finnish women. J Clin Med. 2020;9(5):1588. 32456169 10.3390/jcm9051588 PMC7290663

[23] Jang S-Y, Park J, Ryu S-Y, Choi S-W. Low muscle mass is associated with osteoporosis: a nationwide population-based study. Maturitas. 2020;133(1):54-59. 32005424 10.1016/j.maturitas.2020.01.003

[24] Papageorgiou M, Sathyapalan T, Schutte R. Muscle mass measures and incident osteoporosis in a large cohort of postmenopausal women. J Cachexia Sarcopenia Muscle. 2019;10(1):131-139. 30398016 10.1002/jcsm.12359 PMC6438341

[25] Kapuš O, Gába A, Lehnert M. Relationships between bone mineral density, body composition, and isokinetic strength in postmenopausal women. Bone Rep. 2020;12:100255. 32181269 10.1016/j.bonr.2020.100255 PMC7063090

[26] da Cruz GF, Lunz TM, de Jesus TR, et al. Influence of the appendicular skeletal muscle mass index on the bone mineral density of postmenopausal women. BMC Musculoskelet Disord. 2021;22(1):861. 34627216 10.1186/s12891-021-04748-x PMC8501937

[27] Fighera TM, Santos BR, Motta L, Casanova G, Spritzer PM. Associations between bone mass, hormone levels, and body composition in postmenopausal women. Menopause. 2023;30(3):317-322. 36729603 10.1097/GME.0000000000002126

[28] Gnudi S, Sitta E, Fiumi N. Relationship between body composition and bone mineral density in women with and without osteoporosis: relative contribution of lean and fat mass. J Bone Miner Metab. 2007;25(5):326-332. 17704998 10.1007/s00774-007-0758-8

[29] Ijuin M, Douchi T, Matsuo T, Yamamoto S, Uto H, Nagata Y. Difference in the effects of body composition on bone mineral density between pre- and postmenopausal women. Maturitas. 2002;43(4):239-244. 12468131 10.1016/s0378-5122(02)00273-6

[30] Mizuma N, Mizuma M, Yoshinaga M, et al. Difference in the relative contribution of lean and fat mass components to bone mineral density with generation. J Obstet Gynaecol Res. 2006;32(2):184-189. 16594922 10.1111/j.1447-0756.2006.00384.x

[31] Shieh A, Karlamangla AS, Karvonen-Guttierez CA, Greendale GA. Menopause-related changes in body composition are associated with subsequent bone mineral density and fractures: study of Women’s health across the nation. J Bone Miner Res. 2023;38(3):395-402. 36542065 10.1002/jbmr.4759 PMC10023299

[32] Greendale GA, Sowers M, Han W, et al. Bone mineral density loss in relation to the final menstrual period in a multiethnic cohort: results from the study of Women’s health across the nation (SWAN). J Bone Miner Res. 2012;27(1):111-118. 21976317 10.1002/jbmr.534 PMC3378821

[33] Trajanoska K, Rivadeneira F, Kiel DP, Karasik D. Genetics of bone and muscle interactions in humans. Curr Osteoporos Rep. 2019;17(2):86-95. 30820831 10.1007/s11914-019-00505-1 PMC6424938

[34] Gomarasca M, Banfi G, Lombardi G. Myokines: The endocrine coupling of skeletal muscle and bone. Adv Clin Chem. 2020;94:155-218. 31952571 10.1016/bs.acc.2019.07.010

[35] Chen R, Armamento-Villareal R. Obesity and skeletal fragility. J Clin Endocrinol Metab. 2024;109(2):e466-e477. 37440585 10.1210/clinem/dgad415 PMC10795939

[36] Zhu K, Hunter M, James A, Lim EM, Walsh JP. Associations between body mass index, lean and fat body mass and bone mineral density in middle-aged Australians: the Busselton healthy ageing study. Bone. 2015;74:146-152. 25652209 10.1016/j.bone.2015.01.015

[37] Zhu K, Hunter M, James A, Lim EM, Walsh JP. Relationships between longitudinal changes in body composition and bone mineral density in middle-to-older aged Australians. Osteoporos Int. 2023;34(9):1601-1611. 37233793 10.1007/s00198-023-06773-z PMC10427547

[38] Marlatt KL, Pitynski-Miller DR, Gavin KM, et al. Body composition and cardiometabolic health across the menopause transition. Obesity. 2022;30(1):14-27. 34932890 10.1002/oby.23289 PMC8972960

[39] Seimon RV, Wild-Taylor AL, Keating SE, et al. Effect of weight loss via severe vs moderate energy restriction on lean mass and body composition among postmenopausal women with obesity. JAMA Netw Open. 2019;2(10):e1913733. 31664441 10.1001/jamanetworkopen.2019.13733 PMC6824325

