# Supplementary material for: Associations between body composition and bone loss in early postmenopausal women
Source: J Bone Miner Res. 2025 Sep 11;41(3):251–8. doi: 10.1093/jbmr/zjaf125 (PMC13016812; doi:10.1093/jbmr/zjaf125)
Supplement: Supplementary_Material_zjaf125 [file supplementary_material_zjaf125.docx]

**Supplementary Material**

Geraldi MV, Gregori G, Johansson L, Hjertonsson U, Brättemark E, Lorentzon M. Associations between body composition and bone loss in early postmenopausal women

**Supplemental Table 1.** Analyses of the relative change in lifestyle and bone characteristics between the randomized probiotic treatments and placebo groups.

**Supplemental Table 2.** Interaction p-values between visit and each body composition variable (baseline and change from baseline) vs. each bone outcome.

**Supplemental Figure 1**. Categorization of body composition groups by tertiles of fat mass and ∆% ALM.

**Supplemental Table 1.** Analyses of the relative change in lifestyle and bone characteristics of the according to group of probiotic treatment.

|  | ***Limosilactobacillus reuteri*** | | | |
| --- | --- | --- | --- | --- |
| **Characteristics** | **High dose** | **Low dose** | **Placebo** | **P-value** |
| *Bone mineral density and microstructure* | |  |  |  |
| Total hip BMD, g/cm^2^ | -2.05 (4.67) | -2.60 (3.06) | -1.99 (2.17) | 0.87 |
| Lumbar spine BMD, g/cm^2^ | -1.76 (5.31) | -2.81 (4.05) | -2.07 (2.60) | 0.65 |
| Tibia total vBMD, mg/cm^3^ | -2.66 (3.76) | -2.14 (3.34) | -2.07 (3.04) | 0.94 |
| Tibia trabecular BV/TV, % | -0.63 (2.31) | -1.45 (5.20) | -1.27 (3.27) | 0.39 |
| Tibia cortical vBMD, mg/cm^3^ | -1.84 (2.35) | -1.43 (2.02) | -0.89 (1.37) | 0.32 |
| Tibia cortical area, mm | -4.36 (4.87) | -3.53 (4.53) | -2.71 (4.06) | 0.72 |

Values are expressed as median (IQR) or mean ± SD. P values were obtained using one-way ANOVA to determine statistical significance between groups (p<0.05).

#

# **Supplemental Table 2.** Interaction p-values between visit and each body composition variable (baseline and change from baseline) vs each bone outcome.

| **Outcome** | **Body weight** | | **BMI** | | **Fat mass** | | **ALM** | |
| --- | --- | --- | --- | --- | --- | --- | --- | --- |
|  | **BL × visit**  **Interaction p-value** | **CHG × visit**  **Interaction p-value** | **BL × visit**  **Interaction p-value** | **CHG × visit**  **Interaction p-value** | **BL × visit**  **Interaction p-value** | **CHG × visit**  **Interaction p-value** | **BL × visit**  **Interaction p-value** | **CHG × visit**  **Interaction p-value** |
| Total hip BMD | 0.37 | **0.039** | 0.27 | **0.050** | 0.14 | 0.20 | 0.84 | **<.0001** |
| Neck hip BMD | 0.61 | 0.32 | 0.39 | 0.21 | 0.27 | 0.89 | 0.70 | **0.0017** |
| Lumbar spine | 0.45 | 0.29 | 0.18 | 0.40 | 0.25 | 0.52 | 0.56 | 0.07 |
| Total vBMD | **0.0014** | 0.09 | **0.010** | 0.06 | **0.0005** | 0.13 | 0.15 | 0.17 |
| Trabecular | 0.80 | 0.12 | 0.71 | 0.06 | 0.22 | 0.10 | 0.16 | 0.23 |
| Cortical vBMD | **0.0009** | 0.25 | **0.0010** | 0.30 | **0.0022** | 0.30 | **0.0003** | 0.73 |
| Cortical area | **0.012** | **0.0083** | **0.012** | **0.0088** | **0.0048** | **0.037** | 0.06 | **0.020** |

BL = Baseline, CHG = Change from baseline.

Interaction p-values between visit and each body composition variable per each bone outcome (baseline and change from baseline). Mixed models for repeated measures (MMRM) were used including the interaction between each of the body composition explanatory variables and visit, with an unstructured covariance matrix. The models are adjusted for age, smoking, years since menopause, menopausal hormone therapy use, height, visit, treatment group and the baseline value of each respective outcome.

Abbreviations: BMD, bone mineral density; BMI, body mass index; BV/TV, trabecular bone volume to total volume fraction; vBMD, volumetric BMD. Bone measurements units: total hip BMD, g/cm^2^; neck hip BMD, g/cm^2^; lumbar spine BMD, g/cm^2^; tibia total vBMD, g/cm^3^; tibia trabecular BV/TV, %; tibia cortical vBMD, g/cm^3^; tibia cortical area, mm^2^.

**Supplemental Figure 1**. Categorization of body composition groups by tertiles of fat mass and ∆% ALM.


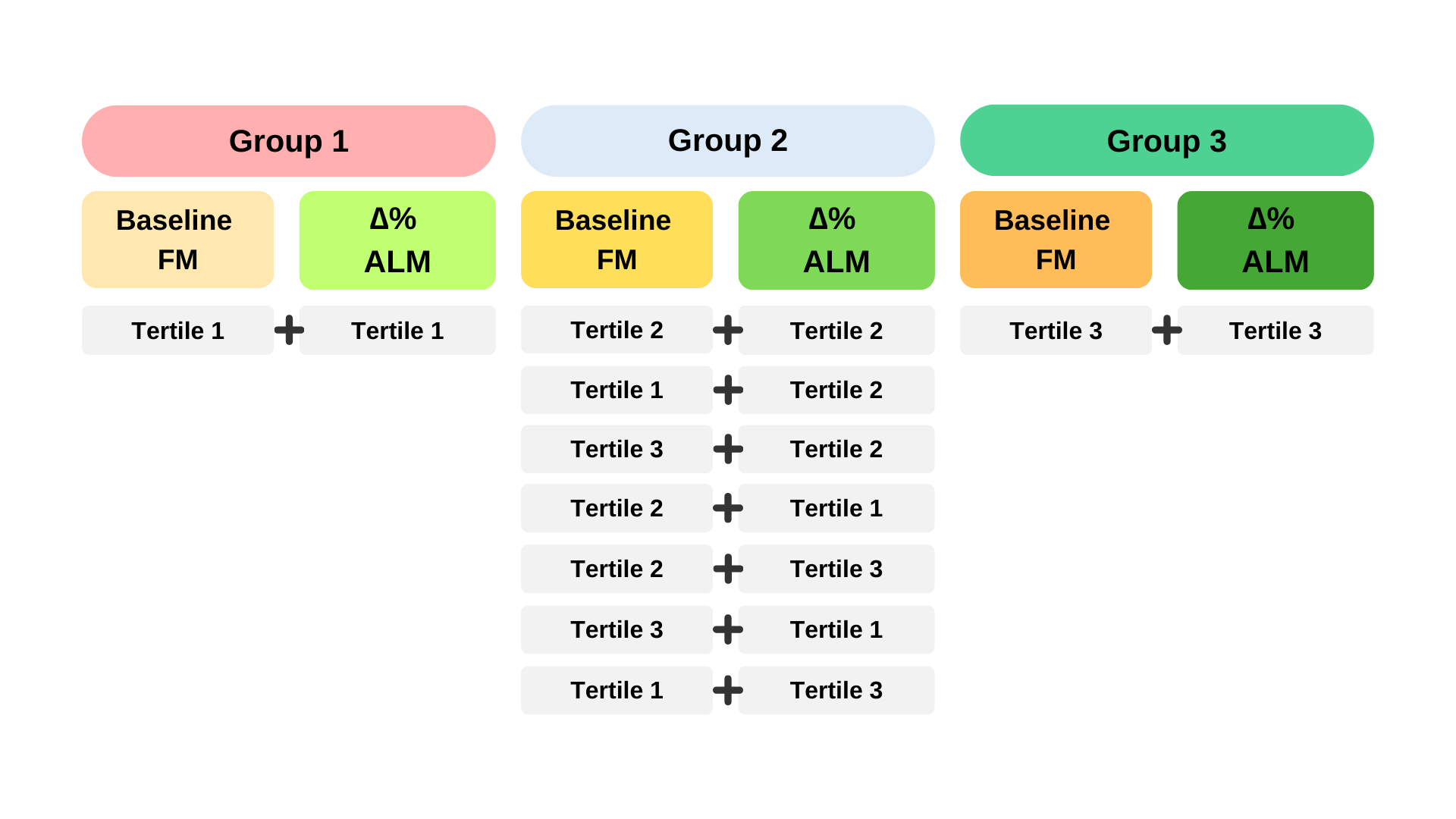


**Figure legend.** ∆%, relative change; ALM, Appendicular lean mass; FM, fat mass.

Participants were ranked into tertiles based on baseline FM and ∆%ALM and categorized into three groups: two extreme groups representing higher and lower tertiles of baseline FM and ∆%ALM, and an intermediate group comprising mixed combinations of FM and ∆%ALM.

- **Group 1**: Participants in tertile 1 for both baseline FM and ∆% ALM.
- **Group 2**: Participants in tertile 2 for both variables or those with mixed combinations, including, tertile 1 for baseline FM and tertile 3 for ∆% ALM, or tertile 3 for baseline FM and tertile 1 for ∆% ALM.
- **Group 3**: Participants in tertile 3 for both baseline FM and ∆%
